# Effect of Total Crown and Veneer Zirconia Meso-Structure Designs on Anterior Implant-Supported Restorations: A 3D Finite Element Analysis

**DOI:** 10.4317/jced.63753

**Published:** 2026-03-30

**Authors:** Guilherme Schmitt de Andrade, Luis Alberto Formighieri, Gabriela Carrão Aragonez, Marina Gullo Augusto, João Paulo Mendes Tribst

**Affiliations:** 1 DDS, MS, PhD. Private Dental Practice, Cascavel, Paraná, Brazil; 2 DDS, MS, PhD. Associate Professor, Dentistry Program, State University of Western Paraná (UNIOESTE), Cascavel, Paraná, Brazil; 3 PhD Student, Post-Graduate Program in Oral Sciences, Prosthodontics Unit, Faculty of Dentistry, Federal University of Santa Maria (UFSM), Santa Maria, Rio Grande do Sul, Brazil; 4 DDS, MS, PhD. Private Dental Practice, Cascavel, Paraná, Brazil; 5 Associate Professor, Department of Reconstructive Oral Care, Academic Centre for Dentistry Amsterdam (ACTA), Universiteit van Amsterdam and Vrije Universiteit, Amsterdam, The Netherlands

## Abstract

**Background:**

Unfavorable implant positioning in esthetic zones may result in buccal or incisal emergence of the prosthetic screw access, requiring alternative restorative strategies. This study evaluated, by three-dimensional finite element analysis (FEA), the effect of total crown and laminated veneer zirconia meso-structure designs cemented onto a titanium base (Ti-base) on stress distribution and bone microstrain.

**Material and Methods:**

Two zirconia meso-structure designs: total crown (TC) and laminated veneer (LV) were simulated on a Ti-base using previously validated models. A 20 Ncm screw torque and a 150 N oblique load at 30° were applied. Stress was analyzed using von Mises and maximum principal stress, and bone response by microstrain.

**Results:**

Both designs showed similar stress distribution in the implant, Ti-base, and screw. Microstrain concentrated mainly in the peri-implant cortical bone, with slightly wider distribution in the LV model.

**Conclusions:**

TC and LV meso-structures exhibited comparable biomechanical behavior, suggesting that their selection may be guided primarily by restorative and esthetic considerations.

## Introduction

Aesthetics has become a fundamental requirement in dental prosthetic planning. In implant-supported restorations placed in esthetic zones, long-term success relies on the appropriate integration of three-dimensional implant positioning, soft tissue management, biomimetic prosthetic design, and a favorable biomechanical response of the restoration (1 , 2). In this context, accurate three-dimensional implant positioning before implant placement surgery is essential to achieve an aesthetically satisfactory final prosthetic result (3). However, when implant placement is guided exclusively by the available bone volume rather than by prosthetically driven planning (backward planning), unfavorable implant positioning may occur, increasing the risk of compromised emergence profile, inadequate soft tissue support, and limited compatibility with conventional prosthetic designs (4). However, in some cases of severely limited bone volume, ideal implant positioning, particularly that which allows for a non-visible screw access channel, may not be feasible (5). Poorly positioned implants are often characterized by an unfavorable relationship between the screw access channel and adjacent teeth and/or alveolar bone, as well as excessive buccal or lingual restorative material volume, ultimately compromising the esthetic outcome of the prosthetic restoration (6). Under such circumstances, alternative strategies are required to overcome these limitations. In these situations, particularly when the screw access channel is buccally positioned, the use of angled abutments combined with cement-retained crowns has been proposed as a means of masking the screw access opening (5). Nevertheless, previous studies have demonstrated that angled abutments may increase stress concentrations within the implant-abutment complex, including the prosthetic screw and the surrounding bone tissue (7 - 10). With the extensive adoption of Computer-Aided Design/Computer-Aided Manufacturing (CAD/CAM) systems in dentistry, hybrid abutments, commonly referred to as titanium bases (Ti-bases), have emerged as a viable alternative to angled abutments (11). These components combine a ceramic meso-structure, most commonly zirconia, with a titanium connection, allowing improved esthetic outcomes through adequate gingival contouring and a favorable peri-implant emergence profile (11 , 12). Moreover, Carvalho et al. verified, through three-dimensional finite element analysis (FEA), that hybrid abutments exhibit superior biomechanical behavior compared with monolithic zirconia abutments, characterized by reduced stress concentration in the ceramic component while preserving the mechanical advantages of titanium at the implant-abutment interface, without compromising the esthetic benefits of ceramic materials (12). The meso-structure associated with hybrid abutments may be fabricated using different prosthetic designs, most notably a total crown, but also laminated veneer preparations. In the total crown design, the restoration typically consists of a full-coverage ceramic crown, such as lithium disilicate (11). However, a limitation of this approach is related to intraoral cementation, as the extrusion of resin cement into the peri-implant sulcus may trigger an inflammatory response, potentially compromising peri-implant soft tissue stability and the maintenance of marginal bone levels (11 , 13). As an alternative, laminated veneer preparation designs have been proposed, as they position the cervical margin at a supragingival level, thereby reducing the risk of subgingival cement extrusion (14). Clinically, this approach has been used to esthetically harmonize restorations involving substrates with different colors and rehabilitation modalities, including tooth-supported total crowns, laminated veneers, metal cores, darkened substrates, and osseo-integrated implants (14 , 15). Although these different designs present distinct clinical and biological implications, their effects on the biomechanical behavior of zirconia meso-structures remain insufficiently elucidated. Despite some proposed implant-supported infrastructure designs (12 , 16), limited information is available regarding stress concentration and distribution in zirconia meso-structures associated with total crown or with laminated veneer preparations, particularly in anterior implant-supported single restorations. In this context, finite element analysis (FEA) emerges as a noninvasive computational method widely used in dentistry to investigate stress distribution and biomechanical behavior of implant-supported restorations under controlled loading conditions (17). This method allows the simulation of complex anatomical structures, material properties, and different prosthetic designs, providing relevant insights into load transfer mechanisms within the implant-abutment-bone complex. Accordingly, FEA enables the comparison of different restorative configurations, contributing to a more comprehensive understanding of the biomechanical behavior of implant-supported rehabilitations (17). Therefore, given the limited evidence regarding stress concentration in zirconia meso-structures associated with different restorative preparation designs, this study aimed to evaluate, by means of 3D finite element analysis, the effect of total crown and laminated veneer meso-structure designs on stress distribution and microdeformation in anterior implant-supported single restorations.

## Materials and Methods

**Figure 1:**
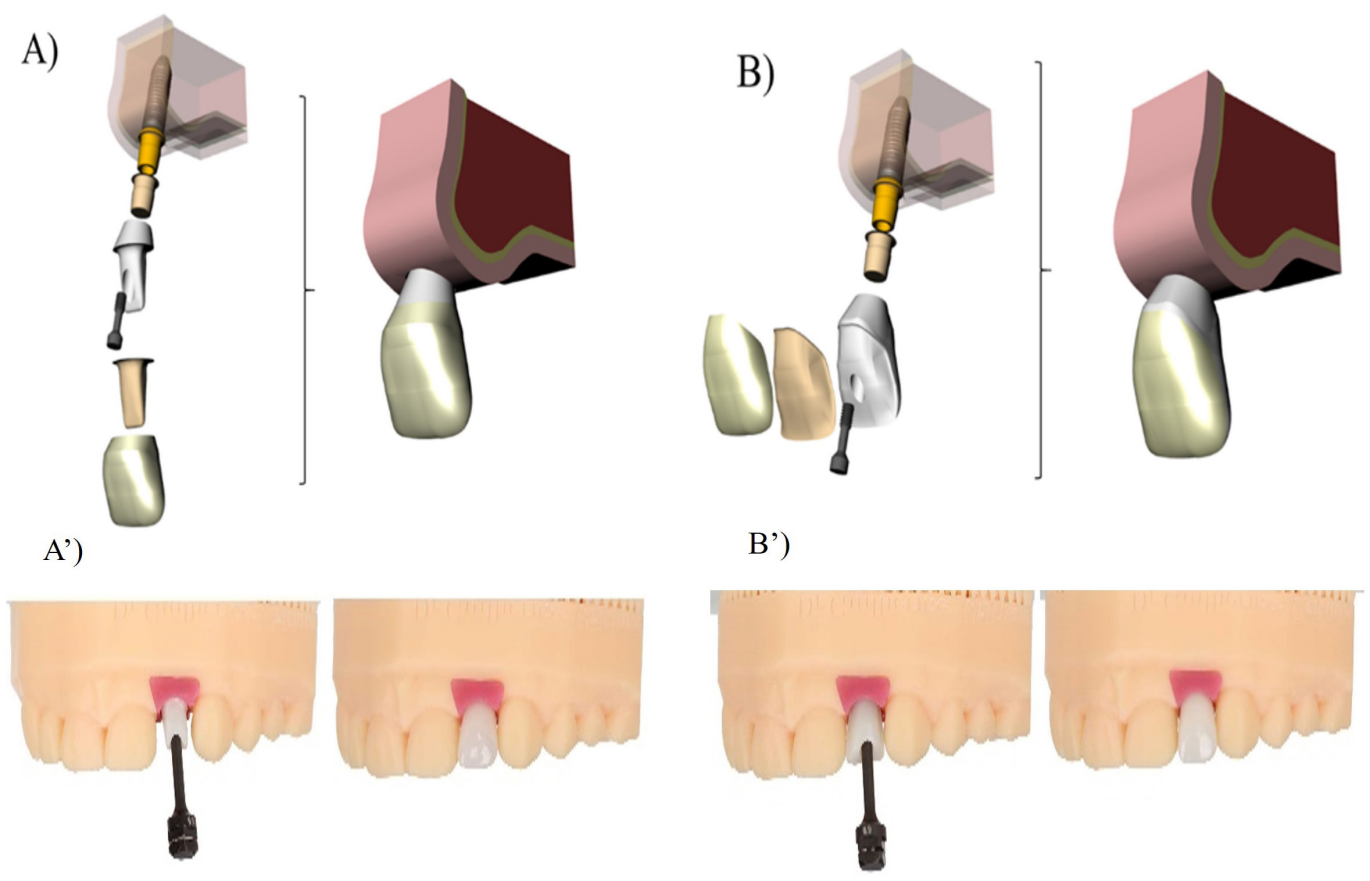
Three-dimensional models illustrating the restorative preparations are shown above: (A) total crown (TC) and (B) laminated veneer (LV). The corresponding preparations performed on a mannequin are shown below: (A’) TC and (B’) LV. The brown three-dimensional structure represented beneath the crown and veneer corresponds to a simulated 80 µm cement layer.

The study was conducted using three-dimensional finite element analysis. Previously published and validated three-dimensional models described by Tribst et al. and Borges et al. were used (18 , 19). These models were imported into a modeling software based on the Non-Uniform Rational B-Spline (NURBS) language (Rhinoceros 5.0; McNeel North America, Seattle, USA) and adapted according to the experimental groups evaluated in the present study, enabling the performance of structural mechanical analysis. A schematic overview of the adopted procedures is presented in Figure 1. Previously published and validated three-dimensional models of a conical connection implant (3.75 × 11 mm), according to the manufacturer's specifications (Conexão Sistemas de Prótese, Arujá, SP, Brazil), a Ti-base (NP model, 4.5 × 5 mm), and a prosthetic screw were used for stress distribution analysis (18). The morphology of the cortical bone, cancellous bone, and the anatomy of the maxillary central incisor crown were based on a previously validated model reported in the literature (19). Using a sound maxillary central incisor as a reference, the preparations of the cemented zirconia meso-structures were modeled. The preparations were defined according to two experimental groups (Figure 1A and 1B, respectively):

1

**Figure 2:**
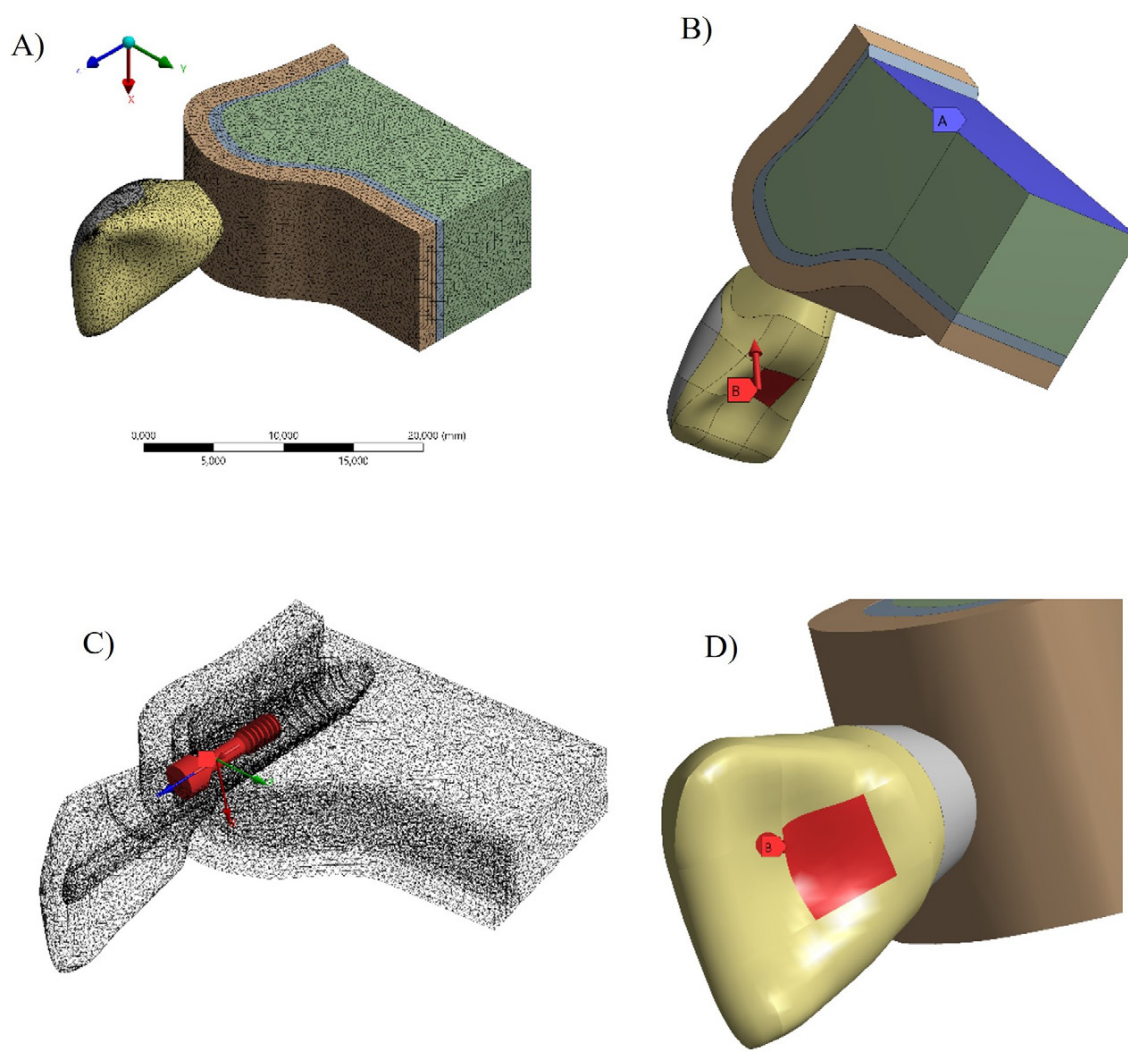
Finite element analysis setup: (A) tetrahedral mesh generation; (B) boundary conditions defined by fixation at the cortical and medullary bone base; (C) tightening torque applied to the prosthetic screw; and (D) loading area.

Total Crown (TC): a lithium disilicate ceramic total crown adhesively cemented onto a hybrid zirconia abutment, prepared with a 1 mm deep chamfer finish line, 1 mm reduction on the buccal, palatal, and proximal surfaces, and a 2 mm incisal reduction. Laminated Veneer (LV): a lithium disilicate ceramic laminated veneer adhesively cemented onto a hybrid zirconia abutment, prepared with a 1 mm deep bevel finish line, 1 mm reduction on the buccal surface, and a 2 mm incisal reduction at 45°. The external surface of each preparation design was duplicated and extruded to generate a cement layer with a uniform thickness of 80 m. After model generation and adaptation, the geometries were exported in Standard for the Exchange of Product Data (STEP) format and imported into computer-aided engineering software (CAE- ANSYS 19.2; ANSYS Inc., TX, United States), where the finite element analyses were performed. Meshes were generated using tetrahedral elements, and a convergence test was conducted to ensure that the number of nodes and elements did not influence the simulation outcomes (Fig. 2A), following methodologies described in previous studies (18 , 19).

2

All materials were considered homogeneous, isotropic, and linearly elastic, in accordance with assumptions commonly adopted in finite element analyses applied to restorative dentistry. The mechanical properties of the materials, including elastic modulus and Poisson's ratio, along with their respective references, are presented in Table 1 (18 , 22 - 25).

Table 1

Boundary conditions were defined by fixing the superior base of the cortical and cancellous bone models in all directions, restricting displacements along the X, Y, and Z axes (Fig. 2B). To simulate the clinical condition of prosthetic installation, a tightening torque of 20 Ncm was applied to the prosthetic screw, consistent with recommended clinical protocols (Fig. 2C). Subsequently, an oblique load of 150 N, applied at 30°, was directed to the cingulum region of the crown to simulate the maximum intercuspation condition (Fig. 2D), as described in previous biomechanical investigations (20 , 21). The numerical results were evaluated based on the stress and strain distributions generated under the applied loading conditions. Stress distribution in the restorative components and cement layer was analyzed using the von Mises equivalent stress and maximum principal stress criteria, according to the mechanical behavior of each material. For the supporting bone structures, the results were evaluated using the microstrain criterion. Colorimetric stress maps were generated for qualitative visualization, and peak stress values were extracted for quantitative comparison among the experimental groups.

## Results

**Figure 3:**
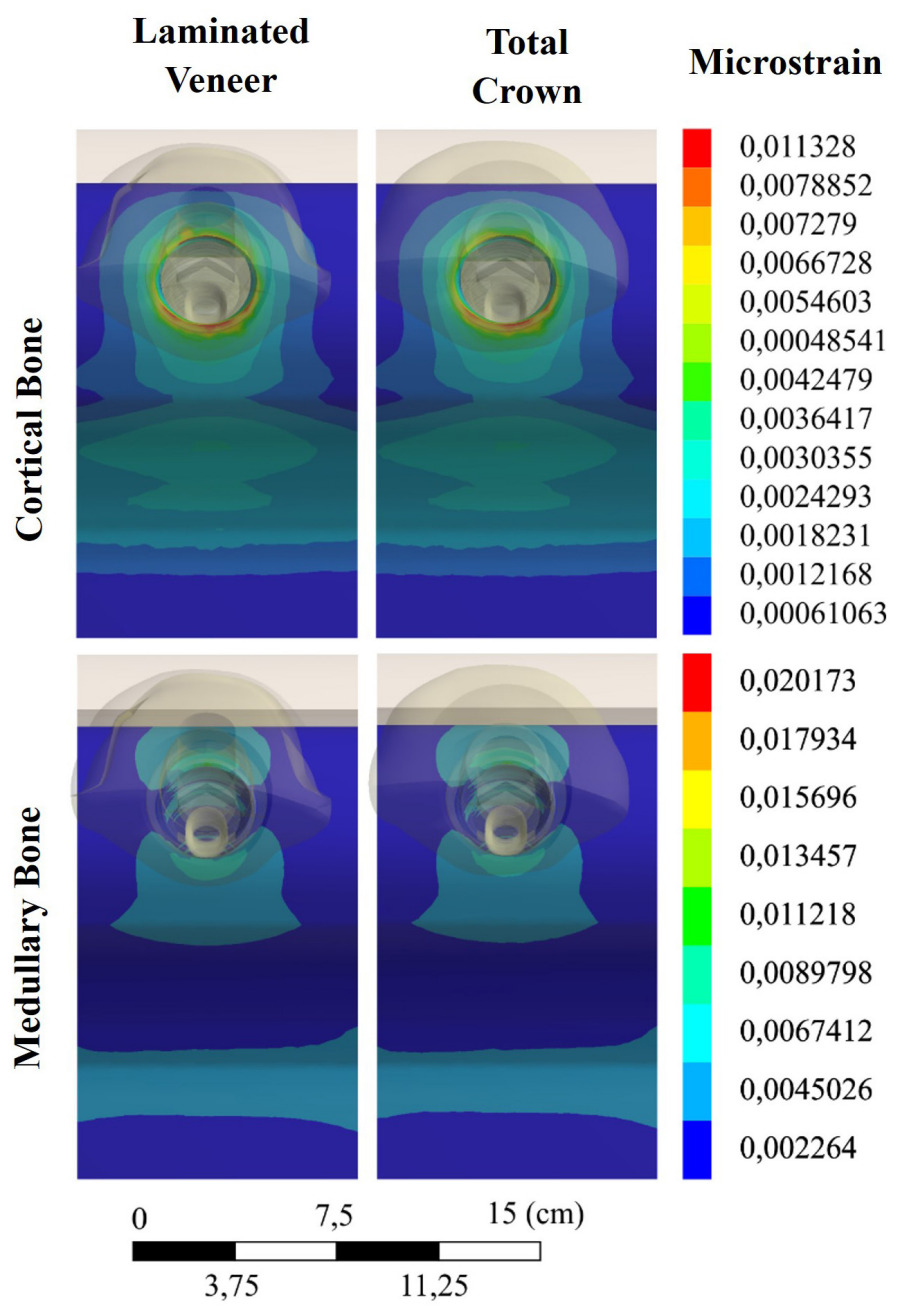
Microstrain distribution in the cortical and medullary bone for the evaluated restorative designs.

The responses of the cortical and cancellous bone are shown in Figure 3.

3

**Figure 4:**
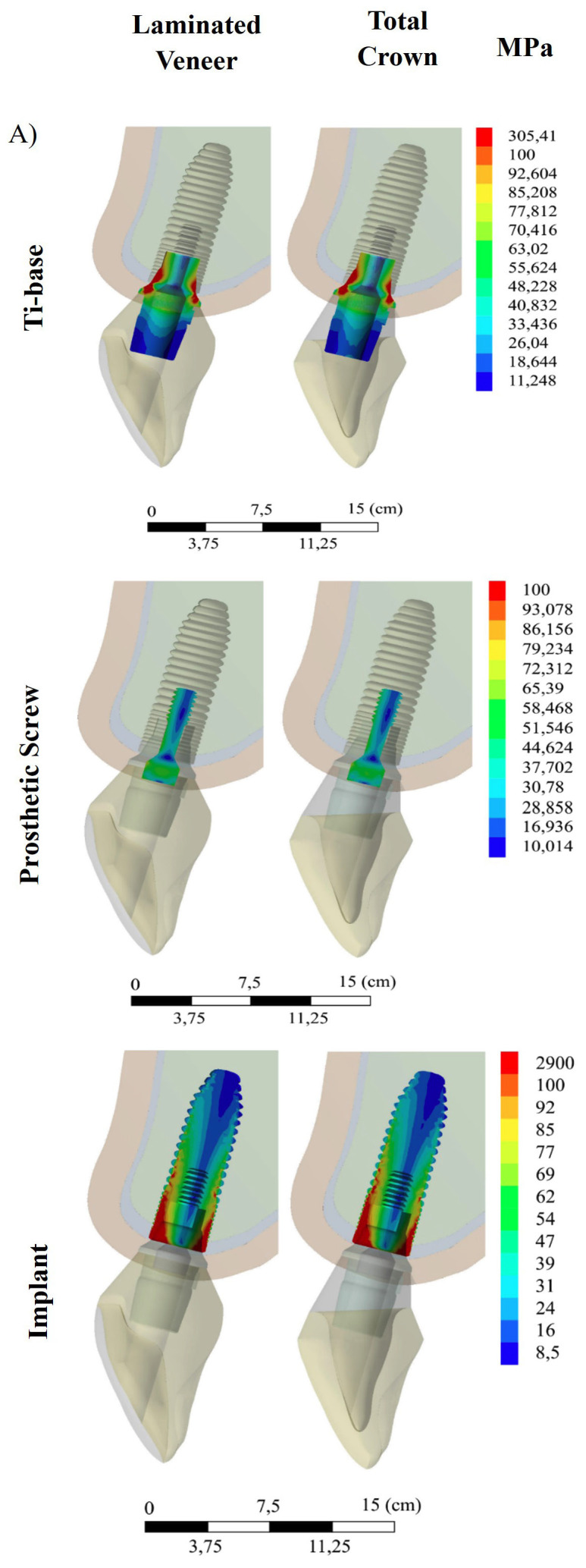
von Mises stress distribution (in MPa) obtained by 3D finite element analysis for the evaluated restorative designs, illustrating stress patterns in the (A) Ti-base, (B) prosthetic screw, and (C) implant.

In both models, microstrain was predominantly concentrated in the cortical bone surrounding the implant neck, forming a characteristic region of higher microstrain values in the peri-implant area. The LV model exhibited a greater extent of regions with elevated microstrain in the cortical bone compared with the TC model. In contrast, the cancellous bone showed a similar distribution and magnitude of microstrain for both restorative designs, with lower levels observed in regions of peri-implant cortical bone. The stress distribution in the Ti-base is presented in Figure 4.

4

For both restorative designs, stresses were mainly concentrated in the coronal portion of the Ti-base, particularly in areas adjacent to the implant-abutment interface and the screw channel, while the remaining regions exhibited lower stress values. The distribution and magnitude of stresses were similar between the evaluated models. Figure 4 presents the results related to the prosthetic screw. In both models, stresses were predominantly located along the screw body and in the head/neck region. The distribution and magnitude of stresses were similar between the LV and TC models, with no relevant differences in the location of stress peaks. The stress distribution in the implant is shown in Figure 4. In both groups, stress concentration was observed in the cervical region and in the first implant threads, particularly on the side corresponding to the direction of the applied oblique load, whereas the more apical regions exhibited lower stress values. The distribution and magnitude of stresses were similar between the LV and TC models.

## Discussion

The present study evaluated, by means of three-dimensional finite element analysis (FEA), the stress and microstrain distribution associated with two zirconia meso-structure designs cemented onto a Ti-base: total crown and laminated veneer configurations. This restorative approach is commonly indicated in situations where implant positioning results in an unfavorable prosthetic screw access trajectory, with emergence in the incisal or buccal region of the restoration. In such cases, the use of hybrid abutments allows preservation of the metal implant-abutment interface while enabling a ceramic superstructure, aiming to combine adequate mechanical performance with esthetic demands. The meso-structure may be designed similarly to a conventional total crown preparation, onto which a glass-ceramic crown is adhesively cemented, a concept represented by the TC group in the present study. However, due to complications associated with cement extrusion, an alternative geometry resembling a laminated veneer preparation has been proposed, originally developed to overcome limitations in restorative space and esthetic interference caused by the screw access channel. Oderich et al. demonstrated that alternative restorative designs cemented onto zirconia abutments can exhibit adequate mechanical performance when combined with appropriate adhesive protocols (15). In the present study, this concept was represented by the LV group. The results revealed similar stress patterns and magnitudes in the Ti-base, prosthetic screw, and implant for both restorative designs (TC and LV). These findings are consistent with previous studies indicating that, in hybrid abutment systems, load transfer is predominantly concentrated at the implant-abutment connection and within the screw assembly, whereas modifications in the coronal geometry of the meso-structure tend to exert limited influence when the connection design, Ti-base, and material properties remain unchanged (18 , 26). Moreover, the Ti-base appears to act as an intermediate component capable of absorbing part of the applied stresses and attenuating their direct transmission to the implant, contributing to similar mechanical behavior across different coronal configurations (27). The bone response followed the expected pattern for oblique loading in anterior implants, with higher microstrain concentrations observed in the peri-implant cortical bone, particularly in the cervical region, while the cancellous bone exhibited lower deformation levels. This behavior is well documented in the literature and reflects the predominant role of cortical bone in load dissipation around osseointegrated implants, especially under oblique forces (28 , 29). FEA studies have also shown that the absence of the periodontal ligament leads to a more direct transfer of loads to the cortical bone, which helps explain the higher microstrain levels observed in this region (17 , 19). Although the LV model exhibited a greater extension of areas with elevated microstrain in the cortical bone, the magnitude and distribution of deformation within the cancellous bone were comparable between the restorative designs. This finding suggests that modifications in the coronal design of the meso-structure exert limited influence on deeper bone responses, if boundary conditions and the implant-abutment connection are maintained, in agreement with previous biomechanical investigations (17 , 29). From a clinical perspective, interest in meso-structure designs that facilitate cement control is also supported by biological complications associated with cement extrusion in implant-supported restorations. Clinical and laboratory studies have demonstrated that residual cement may act as a triggering factor for peri-implant inflammation and peri-implantitis (13 , 30). Although biological aspects were not directly assessed in the present study, the biomechanical feasibility of the LV design observed herein suggests that its indication may be considered when restorative strategies aimed at minimizing cement-related complications are desirable. The results should be interpreted considering the inherent limitations of finite element analysis, including the assumption of homogeneous, isotropic, and linearly elastic material properties, idealized contacts between components, and the absence of fatigue and aging effects. These limitations are widely acknowledged in literature and reinforce the need for complementary laboratory and clinical studies to confirm the present findings under conditions that more closely reflect clinical reality (18 , 19). However, finite element analysis remains a well-established and validated tool for investigating the biomechanical behavior of implant-supported systems, allowing identification of stress and strain distribution patterns and controlled comparisons among restorative configurations. In this context, the present findings contribute to a better understanding of the mechanical performance of different zirconia meso-structure designs and provide relevant support for clinical decision-making and for the direction of future experimental research.

## Conclusions

Within the limitations of this finite element study, both zirconia meso-structures exhibited similar biomechanical behavior with respect to stress distribution in the implant, Ti-base, and prosthetic screw. In both models, higher microstrain concentrations were observed in the peri-implant cortical bone, whereas the cancellous bone showed comparable responses. Therefore, no substantial biomechanical differences were identified between total crown and laminated veneer meso-structures, suggesting that distinctions between these designs are more related to restorative and esthetic considerations than to mechanical behavior.

## Figures and Tables

**Table 1:** Elastic modulus (in Gigapascal - GPa) and Poisson’s ratio of the materials included in the finite element analysis.

Material/structure	Elastic modulus	Poisson’s ratio
Titanium [22]*	112	0.33
Resin Cement [18]	7.5	0.25
Lithium Disilicate [23]	95	0.30
Zirconia [18]	220	0.32
Cortical Bone [24]	13.7	0.33
Cancellous Bone [24]	1.4	0.31
Soft Tissue [25]	1.8	0.30

*The numbers in brackets indicate the references from which the values were obtained.

## Data Availability

The datasets used and/or analyzed during the current study are available from the corresponding author.

## References

[1] Sailer I, Karasan D, Todorovic A, Ligoutsikou M, Pjetursson BE (2022). Prosthetic failures in dental implant therapy. Periodontol 2000.

[2] Nørgaard Petersen F, Jensen SS, Dahl M (2022). Implant treatment after traumatic tooth loss: A systematic review. Dent Traumatol.

[3] Buser D, Martin W, Belser UC (2004). Optimizing esthetics for implant restorations in the anterior maxilla: Anatomic and surgical considerations. Int J Oral Maxillofac Implant.

[4] Alaqeely R, Albaiz A, Alenazi B, Alem M, Alotaibi Y, Alrowis R (2025). Prevalence of Dental Implant Positioning Errors: A Radiographic Analysis. J Clin Med.

[5] Safi Y, Amid R, Zadbin F, Ghazizadeh Ahsaie M, Mortazavi H (2021). The occurrence of dental implant malpositioning and related factors: A cross-sectional cone-beam computed tomography survey. Imaging Sci Dent.

[6] Rasaie V, Abduo J, Falahchai M (2022). Clinical and Laboratory Outcomes of Angled Screw Channel Implant Prostheses: A Systematic Review. Eur J Dent.

[7] Sailer I, Karasan D, Todorovic A, Ligoutsikou M, Pjetursson BE (2022). Prosthetic failures in dental implant therapy. Periodontol 2000.

[8] Tarnow DP, Chu SJ (2021). When to save or remove implants in the smile zone: A clinical report of maxillary lateral incisor implants in malposition. J Esthet Restor Dent.

[9] Sousa MP, Tribst JPM, de Oliveira Dal Piva AM, Borges ALS, de Oliveira S, da Cruz PC (2019). Capacity to Maintain Placement Torque at Removal, Single Load-to-Failure, and Stress Concentration of Straight and Angled Abutments. Int J Periodontics Restorative Dent.

[10] Tribst JP, Rodrigues VA, Dal Piva AO, Borges AL, Nishioka RS (2018). The importance of correct implant positioning and masticatory load direction on a fixed prosthesis. J Clin Exp Dent.

[11] Adolfi D, Tribst JPM, Adolfi M, Dal Piva AMO, Saavedra GSFA, Bottino MA (2020). Lithium Disilicate Crown, Zirconia Hybrid Abutment and Platform Switching to Improve the Esthetics in Anterior Region: A Case Report. Clin Cosmet Investig Dent.

[12] Carvalho MA, Sotto-Maior BS, Del Bel Cury AA, Pessanha Henriques GE (2014). Effect of platform connection and abutment material on stress distribution in single anterior implant-supported restorations: a nonlinear 3-dimensional finite element analysis. J Prosthet Dent.

[13] Burbano M, Valderrama P, Blansett J, Wadhwani CP, Choudhary PK, Rodriguez LC, Rodrigues DC (2015). Characterization of Cement Particles Found in Peri-implantitis-Affected Human Biopsy Specimens. Int J Oral Maxillofac Implants.

[14] Magne P, Magne M, Jovanovic SA (2008). An esthetic solution for single-implant restorations - type III porcelain veneer bonded to a screw-retained custom abutment: a clinical report. J Prosthet Dent.

[15] Oderich E, Boff LL, Cardoso AC, Magne P (2012). Fatigue resistance and failure mode of adhesively restored custom implant zirconia abutments. Clin Oral Implants Res.

[16] Alencar SM, Nogueira LB, Leal de Moura W, Rubo JH, Saymo de Oliveira Silva T, Martins GA, Moura CD (2017). FEA of Peri-Implant Stresses in Fixed Partial Denture Prostheses with Cantilevers. J Prosthodont.

[17] Tribst JPM, Dal Piva AMO, Blom EJ, Kleverlaan CJ, Feilzer AJ (2024). Dental biomechanics of root-analog implants in different bone types. J Prosthet Dent.

[18] Tribst JPM, Dal Piva AMO, Anami LC, Borges ALS, Bottino MA (2019). Influence of implant connection on the stress distribution in restorations performed with hybrid abutments. J Osseointegration.

[19] Borges ALS, Dal Piva AMO, Concílio LRDS, Paes-Junior TJA, Tribst JPM (2020). Mouthguard Use Effect on the Biomechanical Response of an Ankylosed Maxillary Central Incisor during a Traumatic Impact: A 3-Dimensional Finite Element Analysis. Life (Basel).

[20] Di Francesco P, Bechir A, Popescu AI, Chivu MV, Dobrescu AM, Comăneanu RM, Târcolea M (2025). Finite element analysis (FEA) of the stress behavior of some dental materials. J Med Life.

[21] Atif M, Tewari N, Reshikesh M, Chanda A, Mathur VP, Morankar R (2024). Methods and applications of finite element analysis in dental trauma research: A scoping review. Dent Traumatol.

[22] Toparli M (2003). Stress analysis in a post-restored tooth utilizing the finite element method. J Oral Rehabil.

[23] Zarone F, Sorrentino R, Apicella D, Valentino B, Ferrari M, Aversa R, Apicella A (2006). Evaluation of the biomechanical behavior of maxillary central incisors restored by means of endocrowns compared to a natural tooth: a 3D static linear finite elements analysis. Dent Mater.

[24] Carter DR, Hayes WC (1977). Compact bone fatigue damage--I. Residual strength and stiffness. J Biomech.

[25] Holberg C, Heine AK, Geis P, Schwenzer K, Rudzki-Janson I (2005). Three-dimensional soft tissue prediction using finite elements. Part II: clinical application. J Orofac Orthop.

[26] Nokar K, Atri F, Nokar S (2023). Comparative Evaluation of the Effect of Implant Crown Materials on Implant Components and Bone Stress Distribution: A 3D Finite Element Analysis. Int J Dent.

[27] Korkmaz IH, Kul E (2022). Investigation of the Type of Angled Abutment for Anterior Maxillary Implants: A Finite Element Analysis. J Prosthodont.

[28] Talmazov G, Veilleux N, Abdulmajeed A, Bencharit S (2020). Finite element analysis of a one-piece zirconia implant in anterior single tooth implant applications. PLoS One.

[29] Marcián P, Borák L, Valášek J, Kaiser J, Florian Z, Wolff J (2014). Finite element analysis of dental implant loading on atrophic and non-atrophic cancellous and cortical mandibular bone - a feasibility study. J Biomech.

[30] Chee WW, Duncan J, Afshar M, Moshaverinia A (2013). Evaluation of the amount of excess cement around the margins of cement-retained dental implant restorations: the effect of the cement application method. J Prosthet Dent.

